# Damage to Liver and Skeletal Muscles in Marathon Runners During a 100 km Run With Regard to Age and Running Speed

**DOI:** 10.1515/hukin-2015-0010

**Published:** 2015-04-07

**Authors:** Zbigniew Jastrzębski, Małgorzata Żychowska, Łukasz Radzimiński, Anna Konieczna, Jakub Kortas

**Affiliations:** 1 Gdansk University of Physical Education and Sport, Poland.

**Keywords:** blood parameters, long distance running, muscle damage, health risk

## Abstract

The purpose of this study was to determine: (1) whether damage to liver and skeletal muscles occurs during a 100 km run; (2) whether the metabolic response to extreme exertion is related to the age or running speed of the participant; (3) whether it is possible to determine the optimal running speed and distance for long-distance runners’ health by examining biochemical parameters in venous blood. Fourteen experienced male amateur ultra-marathon runners, divided into two age groups, took part in a 100 km run. Blood samples for liver and skeletal muscle damage indexes were collected from the ulnar vein just before the run, after 25, 50, 75 and 100 km, and 24 hours after termination of the run. A considerable increase in alanine aminotransferase (ALT) and aspartate aminotransferase (AST) was observed with the distance covered (p < 0.05), which continued during recovery. An increase in the mean values of lactate dehydrogenase (LDH), creatine kinase (CK) and C-reactive protein (CRP) (p < 0.05) was observed with each sequential course. The biggest differences between the age groups were found for the activity of liver enzymes and LDH after completing 75 km as well as after 24 hours of recovery. It can be concluded that the response to extreme exertion deteriorates with age in terms of the active movement apparatus.

## Introduction

In recent years, the practice of marathons has become very popular. Moreover, much attention has been drawn to the promotion of physical exercise in order to prevent diseases associated with the progress of civilization. Evidence for this tendency was provided by Howley and Franks (1997) considering performance from a health perspective. At the end of the 1990s, in the USA, Bouchard et al. (1990) and Skinner and Oja (1994) presented the H-RF concept (health-related fitness) that assumed health as the aim of fitness development. Such an approach is highly important in terms of individuals as well as the population since in many developing countries the percentage of elderly people in the population is increasing (aging populations). As a result of the promotion of these ideas, the number of physically active people is increasing (mass sport). The data also encompass people who perform extreme forms of recreation such as winter bathing, mountaineering, marathons or ultra-marathons. However, extreme exertion is related to health risk. According to Ayus et al. (2000), there is evidence of hospitalization or even death after completing a marathon run. Despite this, the number of people taking up long-distance running is constantly growing (Mathews et al., 2012). The increased interest in long-distance running among the population has triggered an increased interest among scientists in investigating changes related to performance. Up to now, numerous studies have been conducted on marathon runners; the studied aspects include the factors that influence the time taken to complete the classic marathon run, the economy of used energy, the relation between running speed and VO2max, changes in biochemical indices and, recently, health aspects of exertion (Kratz et al., 2002; McKenzie et al., 1983; Rapoport, 2010; Sjödin and Jacobs, 1981; Smith et al., 2004; Spriet, 2007; Suzuki et al., 2003; Suzuki et al., 2006; Tanaka and Matsura, 1984). Statistical data provided by Mathews et al. (2012) regarding the death rate after completing a marathon run in the USA in the years 2000–2009 revealed 28 fatal cases during the run or within 24 hours after its termination. Deaths occurred mainly among young people, i.e. under 45 years of age. The most commonly used blood indexes that describe health risk in marathon runners during performance and recovery are those that indicate liver damage: aspartate aminotransferase (AST), alanine aminotransferase (ALT) and lactate dehydrogenase (LDH) as well as damage to cardiac or skeletal muscles: LDH, CK (creatine kinase) and CRP (C-reactive protein) (Brancaccio et al., 2007; Kosinski et al., 2007; Lippi et al., 2011; Nuviala et al., 1992; Rumley et al., 1985; Waśkiewicz et al., 2012; Wu et al., 1992). However, there are little data in the literature on this damage after ultra long-distance running (Spiropoulos and Trakada, 2003; Noakes et al., 1983). Other risks for marathon runners are myopathy, which could be the first symptom of Huntington’s disease, or deterioration in arterial condition, which increases the risk of sudden cardiac death (SCD) among men (especially over 38 years of age) (Burr et al., 2012; Roberts et al., 2013). Despite numerous studies, there is no consensus on whether such exertion is beneficial for health, or which factors determine damage to organs. The literature is not precise as to whether age or running speed determines the greatest damage to organs. In order to solve this problem, the authors of this work attempted to answer three main research questions: (1) whether damage to liver and skeletal muscles occurs during a 100 km run; (2) whether the metabolic response to extreme exertion is related to the age or running speed of the participant; (3) whether it is possible to determine the running speed and distance that would be safe for long-distance runners’ health by examining biochemical parameters in venous blood.

## Material and Methods

### Participants

The study protocols received ethical approval from the Ethical Committee of the Regional Medical Chamber in Gdansk. A total of 14 males volunteered for the study (age: 43.36 ± 11.83 years; body height: 175.29 ± 6.98 cm; body mass: 72.12 ± 7.36 kg). All of them were experienced amateur ultra-marathon runners and signed an informed consent form to participate in the study. The subjects were divided into two age groups: younger (group A, 32 ± 5.33 years), and older (group B, 50.56 ± 9.7 years), and into two groups according to their running speed: faster (group A_1_, 2.81 ± 0.18 m/s) and slower (group B_1_, 2.43 ± 0.12 m/s). The selection resulted in equally divided groups, with seven subjects in each. Significant differences were revealed for the age of the subjects (*p* = 0.0009) and running speed (*p* = 0.0004). All of the subjects had valid medical examinations and received supervision during the experiment.

### Procedures

The 100 km run started at 7:30 a.m. and finished (for the last runner) at 7:38 p.m. The subjects repeatedly ran a designated route of 3300 m. The altitude was 20 m above sea level, and the altitude differences did not exceed 3 m. The running surface was made of asphalt. The temperature during the run ranged between 4–7 °C, the wind speed was 0.7–1.4 m/s, and the humidity was 83–89%.

The shortest time required to complete the 100 km run was 9 hours and 11 minutes, and the longest time was 12 hours and 8 minutes (the time did not encompass the 1-minute breaks for collection of blood samples). The run times and break times between the courses were measured with a stopwatch (Timex brand, Switzerland, 2009), and the time results after each turn were shown on an electronic board at the starting line. Running speed was measured individually every 25 km. Each participant wore running shoes, a T-shirt, a tracksuit (a cotton sweatshirt and pants), gloves and a cap (made of natural fibres). The participants’ individual clothing preferences were accepted. Each of the runners was equipped with a portable heart rate monitor (Polar Electro, OY, Finland), which measured the heart rate in 5-second intervals. Twelve hours before the experiment, the subjects ate supper. After a night’s rest, they ate a light breakfast of their choice at 6:30 am. During the run, the runners nourished themselves with prepared food and drinks served at a special stand. The meal included water with a low mineral content, high-energy drinks, sandwiches with cheese or ham, high-energy bars, and bananas.

Blood samples analysed for indexes of liver and muscle damage, corrected for hemoconcentration, were calculated following the indexes of Dill and Costill (1997). The reference values for the tested indexes were ALT (< 38.0 U/l), AST (< 40 U/l), LDH (80–285 U/l), CRP (< 5.0 mg/l), CK (< 160 U/l) (Tomaszewski, 2001). The De Ritis ratio was expressed as the ratio of AST to ALT (Arthi et al., 2011). Blood samples were collected from the ulnar vein just before the run, after 25, 50, 75, and 100 km, as well as 24 hours after termination of the run. Biochemical analyses were conducted with the use of an A-15 analyser (Biosystems SA, Costa-Brava, Barcelona, Spain, 2012).

### Statistical analyses

The results are expressed as mean values and standard deviations. The Shapiro–Wilk test was applied to assess the homogeneity of the dispersion from the normal distribution. The Levene test was used to verify the homogeneity of variance. For homogenous results, analysis of variance (ANOVA) for repeated measures and the post-hoc HSD Tukey test for equal sample sizes were performed to identify significantly different results. For heterogeneous results, the ANOVA Friedman’s test and the right post-hoc test were applied. The significance level was set at *p* < 0.05. The results were analysed using Statistica 9.0 software (Statsoft, Tulsa, Oklahoma). Additionally, the d Cohen effect size was calculated to enable comparison of the results between the groups (older vs. younger, and faster vs. slower).

## Results

During the 100 km run, the mean values for V, HR and % HRmax decreased considerably (p < 0.05) in each of the 25-kilometer stretches among the whole group as well as in the age groups (group A, B) and speed groups (A1, B1). However, there were no relevant differences between the groups in terms of running speed for the consecutive 25-kilometer stretches (Table 1).

The activity of the liver enzymes ALT and AST corresponded with reference values at rest. However, a considerable increase was observed with the distance covered (p <0.05), which continued during recovery. The standard deviation value was higher during recovery than during exertion. The value of the De Ritis ratio increased significantly with the distance covered, and substantially decreased after 24 hours of rest. There was a considerable increase in the mean values of LDH, CK and CRP (p < 0.05) with each sequential course. During recovery, a significant increase in these indexes was observed in relation to values at rest or during the run (Table 2).

**Figure 1:**
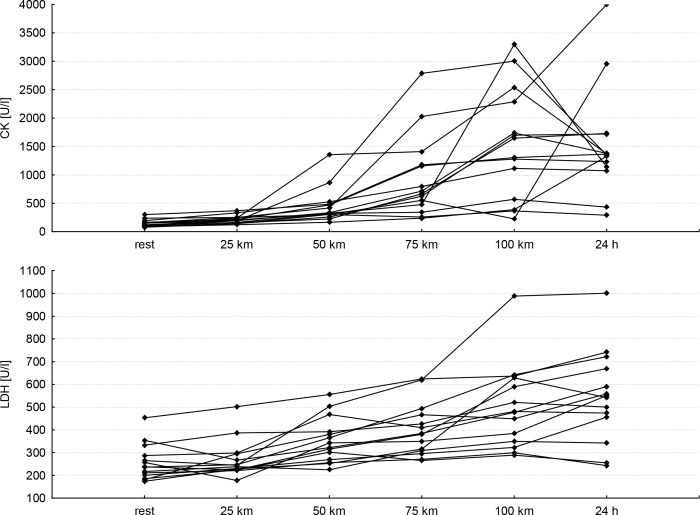
Individual changes in creatine kinase (CK) and lactate dehydrogenase (LDH) during the 100-km run and 24 hours of recovery

The biggest differences found for the activity of liver enzymes and LDH between the age groups were revealed after completing 75 km of the run as well as after 24 hours of recovery (older subjects reached higher values of these indexes). CK activity among the younger group was lower during the run, up to 75 km. Individual changes in CK and LDH activity in both tested groups showed high diversity. The reason for this may be the varied response of individual athletes to the extreme effort as well as different exercise capacity, degree of training, etc. Furthermore, a similar direction of changes in individual CK and LDH was observed. In the majority of respondents, a significant increase in the enzymes was observed 24 hours after termination of exercise (Figure 1). The CRP protein concentration was higher among younger subjects during the entire distance and recovery (Table 3). Higher values of indexes for liver and skeletal muscle damage were found in the faster group during both the run and recovery (Table 4)

## Discussion

The results revealed substantial changes in the indexes of liver and skeletal muscle damage in ultra-marathon runners (*n* = 14) during the 100 km run and after 24 hours of recovery (Table 2). Advanced age and higher running speed appeared to be the factors that increased the activity of liver enzymes and led to muscle damage. The present study supports findings from previous investigations (Rumley et al., 1985; Waśkiewicz et al., 2012; Whitfield, 2001; Rosales et al., 2008). Waśkiewicz et al. (2012) reported a 14-fold increase in AST activity and a four-fold increase in ALT activity within 24 hours of running a marathon. Lippi et al. (2011) traced AST and ALT changes during a half-marathon run and found liver damage, which suggests that such exertion is at variance with health recommendations. However, Smith et al. (2004) and Wu et al. (2004) observed only a slight increase in liver markers in marathon runners. According to Noakes et al. (1983), Whitfield (2001) and Rosales et al. (2008), an increase in AST and ALT activity as an effect of prolonged exertion can indicate considerable damage to liver and skeletal muscles (Noakes et al., 1983; Whitfield, 2001; Rosales et al., 2008). Our study revealed that the highest level of liver enzymes activity occurred 24 hours after termination of the run. However, a considerable increase could be observed after the 50^th^ kilometre of the run; thus, exertion beyond this distance can generate toxic damage to the liver or muscle, as demonstrated by the De Ritis ratio.

The activity of LDH and CK enzymes is commonly used as a marker that indirectly indicates damage to skeletal muscles (Noakes et al., 1983; Kim et al., 2007; Kim et al., 2009; Miles et al., 2008; Brancaccio et al., 2010). In our study, an increase in the activity of these enzymes was observed in the ultra-marathon runners during exertion and recovery. Moreover, even a 10-fold increase in CK activity was revealed (Table 2). However, Kłapcińska et al. (2013) reported a 20-fold increase in the activity of this enzyme 24 hours after completing the ultra-marathon run. Smith et al. (2004) and Wu et al. (2004) also found considerable changes in the activity of CK, ASP and LDH during marathon running and focused on the time needed to attain the balance of enzyme activity. Additionally, Lippi et al. (2011) reported increased activity of LDH and CK during half-marathon running in all studied subjects, especially 24 hours after the termination of the run, when an acute metabolic response occurred. Therefore, the literature suggests the need for monitoring CK as the first sign of asymptomatic myopathy (Kosiński et al., 2007; Brancaccio et al., 2007). High values of CK 24 h after a run were previously observed by Weight et al. (1991) and Chatzinikolaou (2010). Moreover, during the night rest, the blood pressure and heart rate lowered (Table 2, Figure 1).

Mean CRP levels exceeded the reference values in subjects after 75 km and a further increase to 15-fold was observed during recovery in relation to values at rest (Table 2). Shin and Lee (2012) found similar results for CPR values in marathon runners. Therefore, it could be concluded that long-term exertion such as marathon running induces acute systemic inflammation, which is harmful to health.

Although previous studies traced changes in the indexes of liver and skeletal muscle damage, only a few showed results with respect to age or running speed (Ayus et al., 2000; Mathews et al., 2012; Burr et al., 2012). These studies also reported a higher risk of death, including SCD, or a negative effect on arteries among ultra-marathon runners. Roberts et al. (2013) compared the response to long-term exertion in relation to gender and found that male runners were at a higher risk of SCD (especially males aged over 38 years) than female participants. However, the role of age in determining the risk of health changes associated with indexes of liver and skeletal muscle damage remains unknown. Our results reveal a varied response to ultra-marathon runners depending on age. Older subjects showed overactivity of liver enzymes later than younger runners. However, their ALT, AST, the De Ritis and LDH ratio attained higher values after 75 km of a run and during recovery (Table 3). The important fact is that no significant differences in running speed were observed between the groups during the entire distance (Table 1). Therefore, we concluded that older runners showed reduced adaptive abilities of liver to long-distance running than younger subjects, although in both groups an adverse response regarding the health of this organ was observed. As for skeletal muscle damage determined by CK activity, a similar response was observed in ultra-marathon runners. Older participants showed higher LDH activity in the final stage of the run and during recovery and higher CK activity during the run, but not at the end and during recovery. Changes in CRP protein indicated that the response of younger subjects was more acute than in older runners during the run and recovery, thus more harmful to health, as the attained values exceeded the reference values by several times (Table 3). Running speed can be a reliable indicator of physiological adaptation to prolonged exertion in marathon runners. Table 4 shows that the subjects who covered the distance of 100 km in a shorter time (though there were no statistically significant differences in speed results between the groups) had lower indexes of liver and muscle damage during the run and recovery. Therefore, we concluded that they possessed better adaptive mechanisms to long-term exertion. The results of the present study suggest that (1) damage to liver and skeletal muscles occurs in ultra-marathon runners during runs over 50 km. (2) Age and running speed are significant factors in liver and muscle damage. Older subjects show a greater response to exertion after the 50th kilometer and during recovery, but faster runners are better adapted to long-term exertion. (3) Judging by the study results, it can be concluded that running at 2.5–3.0 m/s and up to 50 km could be safe in terms of health. Exertion exceeding this distance and running speed can result in harmful biochemical changes. Therefore, monitoring of biochemical indexes of venous blood should be compulsory (until reference values are attained), especially during a recovery period lasting over 24 hours.

## Conclusion

The results of ultra-marathon runners revealed damage to liver and skeletal muscles, as well as acute metabolic responses that occurred 24 hours after the run. With the use of d Cohen, it was determined that the age of the participants affected the response to extreme exertion. Older runners demonstrated higher activity of liver enzymes during the run over 50 km and during recovery. Furthermore, the activity of muscle damage-associated enzymes (especially LDH) was higher than in younger subjects. Therefore, it can be concluded that the response to extreme exertion deteriorates with age in terms of the active movement apparatus. In terms of running speed, faster runners demonstrated better adaptive abilities (lower activity of liver and skeletal muscle enzymes) to long-term exertion. The distance of 100 km appeared to be too long for our participants, and thus harmful to health. However, a distance of 50 km covered at a speed of 2.5–3.0 m/s was assumed to be relatively safe. Monitoring of runners during extreme performance and recovery seems necessary.

## Figures and Tables

**Table 1:** Running speed and the heart rate of the amateur ultra-marathon runners during sequential courses of the 100 km run (^*^p ≤ 0.05)

	V [m/s]				HR [bpm]				% HRmax			

Group	0–25 km	26–50 km	51–75 km	76–100 km	0–25 km	26–50 km	51–75 km	76–100 km	0–25 km	26–50 km	51–75 km	76–100 km
Total	2.9 ± 0.2^*1–3,4^	2.7 ± 0.27^*2–4^	2.6 ± 0.36	2.4 ± 0.3	145 ± 7.5	148 ± 6.55^*2–4^	143 ± 5.78	137 ± 3.89	78 ± 4.27	80.0 ± 3.65^*2–4^	78 ± 2.75	74 ± 2.07
A	3.00 ± 0.15^1–4^	2.81 ± 0.14	2.82 ± 0.12	2.43 ± 0.37	146 ± 8.76	149 ± 8.51	146 ± 6.7	139 ± 3.69	78 ± 6.08	79 ± 5.44	78 ± 4.35	74 ± 3.17
B	2.93 ± 0.18^*1–3,4^	2.71 ± 0.28	2.53 ± 0.3	2.37 ± 0.3	144.3± 7.34	147 ± 5.55	141 ± 4.26	136 ± 3.67	79 ± 3.09	80 ± 2.38	77 ± 1.43	75 ± 1.26
A_1_	3.06 0.15^*1–4^	2.88 ± 0.17	2.83 ± 0.17	2.58 ± 0.26	142 ± 4.69	145 ± 3.67	142 ± 3.02	137 ± 2.68	77 ± 4.45	78 ± 3.69	77 ± 2.95	74 ± 2.53
B_1_	2.88 0.17^*1–3,4^	2.60 ± 0.23^*2–4^	2.38 ± 0.22	2.21 ± 0.26	149 ± 9.33	152 ± 8.41	144 ± 9.0	138 ± 5.56	81 ± 2.15	82 ± 1.79^*2–4^	79 ± 2.35	75 ± 0.5

* significant differences between particular phases of the run

(1 - rest, 2 – 25 km, 3 – 50 km, 4 – 75 km, 5 – 100 km)

**Table 2:** Indexes of liver and skeletal muscle damage in ultra-marathon runners (n = 14) during the 100 km run and 24 hours of recovery

Index	1 Rest	2 25 km	3 50 km	4 75 km	5 100 km	6 24h recovery
ALT [U/l]	24.07± 8.14^*1–7^	24.72 ± 8.68 ^*2–6^	24.51 ± 7.83^*3–6^	26.83± 9.79 ^*4–6^	35.5 ± 15.53	61.37 ± 29.08
AST [U/l]	31.07 ± 6.22^*1–5,6^	39.52 ± 10.46^*2–6^	45.0 ± 10.61^*3–6^	61.06 ± 28.49^*4–6^	117.53 ± 80.11	185.43 ± 119.21
De Ritis ratio [AST/ALT]	1.36 ± 0.29^*1–4,5,6^	1.66 ± 0.29^*2–5,6^	1.90 ± 0.36^*3–5,6^	2.30 ± 0.58^*4–5^	3.17 ± 0.97	2.85 ± 0.75
LDH [U/l]	256.53 ± 78.87^*1–5,6^	270.51 ± 89.23^*2–5,6^	353.5 ± 109.63	400.85 ± 116.62	504.25 ± 187.75	546.2 ± 218.94
CK [U/l]	145.87 ± 99.1^*1–4^	213.21 ± 120.14^*1–4,5,6^	453.83 ± 321.35^*2–5,6^	944.9 ± 814.24	1532.75 ± 981.91	1526.77 ± 1020.82
CRP [mg/l]	2.69 ± 7.07 ^*1–5,6^	3.30± 9.23^*2–6^	3.83 ± 11.26^*3–6^	5.69 ± 15.33^*4–6^	9.08 ± 19.71	30.09 ± 12.95

* significant differences between particular phases of the run

(1 - rest, 2 – 25 km, 3 – 50 km, 4 – 75 km, 5 – 100 km, 6 - after 24 h)

**Table 3:** Differences between the indexes of liver and skeletal muscle damage in ultra-marathon runners between the groups with respect to age (younger group A vs. older group B).

Index	Rest		25 km		50 km		75 km		100 km		24h recovery	

	A	B	A	B	A	B	A	B	A	B	A	B
ALT	24.7±6.31	23.5±9.5	24.9±6.72	24.6±10.18	24.7±6.17	24.4±8.92	26.1±9.54	27.6±10.45	33.3±21.05	37.7±12.2	55.4±40.43	67.3±19.77
ES	0.15		0.03		0.04		−0.16		−0.28		−0.41	

AST	31.7±5.75	30.5±6.8	40.6±4.97	38.5±13.2	42.5±12.61	47.5±9.52	51.1±44.95	71.1±12.21	95.1±124.02	140.0±35.73	144.9±167.05	226.0±65.6
ES	0.20		0.20		−0.47		−0.70		−0.56		−0.68	

DeRitis ratio	1.36±0.26	1.36±0.32	1.67±0.33	1.65±0.32	1.85±0.31	1.95±0.32	2.19±0.2	2.41±0.32	3.02±0.28	3.32±0.34	2.74±0.27	2.96±0.41
ES	0.02		0.10		−0.27		−0.39		−0.30		−0.30	

LDH	277.0±47.57	236.0±93.37	284.4±79.02	256.7±98.41	316.8±115.62	390.2±108.49	396.8±151.87	404.9±102.68	469.5±288.49	539.0±111.53	518.8±325.89	573.6±124.97
ES	0.52		0.31		−0.67		−0.07		−0.37		−0.25	

CK	114.2±148.25	177.6±40.6	177.2±169.61	249.3±70.45	296.4±432.27	611.3±105.88	721.0±1101.43	1168.8±623.63	1699.7±1175.42	1365.8±935.73	2032.1±529	1021.5±1106.61
ES	−0.64		−0.60		−0.98		−0.55		0.34		0.99	

CRP	4.63±0.26	0.75±8.99	5.65±0.28	0.95±11.8	6.76±0.25	0.9±14.39	9.45±0.82	1.93±18.95	13.91±2.75	4.25±24.28	35.4±12.41	24.78±12.24
ES	0.55		0.51		0.52		0.49		0.49		0.82	

ES - effect size

**Table 4:** Differences between the indexes of liver and skeletal muscle damage in ultra-marathon runners between the groups with respect to running speed (fast group A_1_ vs. slow group B_1_).

Index	Rest		25 km		50 km		75 km		100 km		24h recovery	

	A_1_	B_1_	A_1_	B_1_	A_1_	B_1_	A_1_	B_1_	A_1_	B_1_	A_1_	B_1_
ALT	25.1±10.09	22.1±6.41	25.8±10.42	22.8±7.44	26.0±9.65	23.1±6.66	28.6±10.58	25.0±9.38	36.2±14.41	34.8±17.7	64.2±29.3	58.6±31.36
ES	0.37		0.35		0.38		0.37		0.09		0.19	

AST	31.9±5.04	28.7±6.12	40.0±11.88	38.3±10.41	45.6±10.78	42.2±9.95	60.5±20.99	61.7±36.28	101.1±63.2	134.0±96.32	194.0±129.9	176.8±119.1
ES	0.51		0.16		0.32		−0.04		−0.41		0.14	

DeRitis ratio	1.40±0.36	1.33±0.3	1.75±0.3	1.54±0.28	2.12±0.39	1.78±0.38	2.19±0.37	2.46±0.3	3.39±0.3	3.01±0.28	2.86±0.37	2.84±0.34
ES	0.23		0.72		0.95		−0.47		0.40		0.02	

LDH	280.7±89.88	225.1±64.63	293.8±113.97	246.8±25.57	382.1±94.05	298.2±112.64	422.2±125.37	379.5±125.37	507.9±129.88	500.6±243.86	558.4±191.05	534.0±261.9
ES	0.70		0.53		0.77		0.37		0.04		0.11	

CK	141.7±65.47	105.7±26.38	212.1±101.43	166.1±42.83	421.5±242.72	440.9±404.83	1200.0±1036.9	689.8±457.58	1453.8±969.63	1611.7±1065	1553.7±1262.8	1499.8±834.28
ES	0.36		0.38		−0.06		0.63		−0.16		0.05	

CRP	5.04±7.07	0.65±0.69	6.39±9.23	0.61±0.68	7.44±11.26	0.67±0.71	9.67±15.33	1.69±1.48	13.16±19.71	4.99±2.13	22.11±12.95	38.07±12.24
ES	0.62		0.63		0.60		0.52		0.41		0.41	

ES - effect size
